# Interest in computer-assisted surgery on the glenoid implant positioning in the context of navigated or planned total shoulder arthroplasties

**DOI:** 10.1016/j.jseint.2025.06.003

**Published:** 2025-06-30

**Authors:** Yannis Yahiaoui, Cyril Lazerges, Michel Chammas, Bertrand Coulet

**Affiliations:** Upper Limb, Hand, and Peripheral Nerve Surgery Unit, Orthopedic Surgery Department, Lapeyronie University Hospital, Montpellier, France

**Keywords:** Total shoulder arthroplasty, Navigation, Computer-assisted surgery, Reverse total shoulder arthroplasty, Anatomic total shoulder arthroplasty, Glenoid positioning

## Abstract

**Background:**

Three-dimensional planning and intraoperative navigation are beneficial for glenoid implant positioning in total shoulder arthroplasty (TSA). The respective benefits of these two techniques are still being evaluated. The aim of this study was to evaluate the contribution of intraoperative navigation to glenoid implant positioning, compared with planning alone. Our hypothesis is that the use of intraoperative navigation can help to come closer to the planned positioning of the implant, compared with standard instrumentation.

**Methods:**

This monocentric, ongoing study included 205 shoulders (197 patients) operated between 2018 and 2024 for a TSA, anatomic or reverse. All patients benefited from preoperative planning (Equinoxe Planning App; Exactech, Gainesville, FL, USA), enabling the collection of native glenoid parameters. Postoperatively, these were assessed using the same method via the planning software. One hundred fifty-three TSA were included, and we identified 2 groups: 101 navigated TSA (navigated planned arthroplasties [NAV] group) and 52 planned TSA (planned arthroplasties [PLA] group), comparable in all respects (68% women, mean age 72.5 years). Version and inclination were compared, as well as the difference between planned and postoperative.

**Results:**

The average native glenoid parameters measured and planned were similar between the two groups. The average postoperative version was respectively −2.5° (±4.7°) vs. −1.6° (±6.2°) in the NAV group (*P* = .259), and the postoperative inclination was respectively 3.8° (±4.9°) vs. 2.0° (±8.1°) in the NAV group (*P* = .312). The average postoperative deviation from planning in version was 3.0° (±2.9°) in the NAV group vs. 3.7° (±3.0) (*P* = .157). Regarding inclination, the mean deviation was 4.2° (±3.5°) vs. 6.5° (±5.1°) in the PLA group (*P* = .004). There was a reduction in the proportion of mispositioned implant in the NAV group, both in version (6.9% vs. 13.5%) and in inclination (9.9% vs. 19.3%). In the revered TSA subgroup, we found a significant reduction of the postoperative deviation from planning in the NAV group.

**Discussion:**

Our study highlights the benefits of preoperative planning. We found a reduction in the deviation from planning for reverseTSA, as well as a reduction in the proportion of mispositioned implants. Our series is limited by the absence of randomization. Further studies are needed on clinical improvement after navigation-assisted surgery.

Arthroplasty is the second most frequent shoulder surgery after rotator cuff repair. In 2018, there were 17,043 surgical procedures in France for primary implantation of a shoulder arthroplasty, representing 7.3% of all surgeries involving the shoulder. Of these, 16,003 involved the implantation of a total shoulder arthroplasty (TSA), either anatomic (aTSA) or reverse (rTSA).^20^ This procedure is more frequently performed on women than men, and the average age at surgery is 70.^2^

The main aim of arthroplasty is to restore mobility and reduce shoulder pain.

During surgery, implant positioning is a key factor in ensuring a good clinical outcome. Poor implant positioning can lead to postoperative complications.^12^ The positioning of the glenoid implant is complex, due to poor visualization from the approach, glenoid morphological anomalies, and the size of the glenoid.^6^ Three-dimensional (3D) planning, the use of patient-specific instrumentation, and computer-navigated surgery are ways of improving glenoid implant placement during surgery.^19^ The benefit of intraoperative navigation on implant positioning varies from study to study, with some authors finding no significant difference on glenoid version,^1^ while others^10^ suggest better implant positioning.

In view of these findings, the aim of our study is to investigate whether the use of navigation-assisted surgery combined with preoperative 3D planning results in better glenoid implant positioning than 3D planning alone.

## Materials and methods

### Population-group distribution

This retrospective, monocentric, multioperator study covers a consecutive series of patients operated between 2018 and 2024. We included patients operated for a primary implantation of an aTSA or rTSA, after completion of preoperative planning.

All patients were initially scheduled for intraoperative navigation. However, the choice of navigation was made on the day of surgery, independently from the operator, depending on the availability of navigation ancillary equipment, hardware, or software functionality. This enabled the arthroplasties to be divided into 2 distinct groups: navigated planned arthroplasties (NAV), and nonnavigated planned arthroplasties (PLA).

Exclusion criteria included patients with an indication for an associated glenohumeral procedure (osteotomy, muscle transfer, allograft) and absence of preoperative planning for any reason (no preoperative computed tomography [CT] scan, planning impossible).

**Figure 1:**
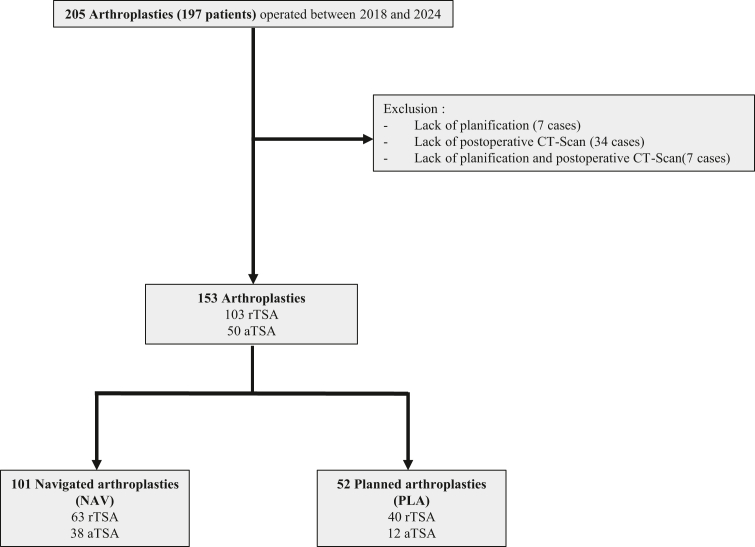
Flowchart, overall population. *CT*, computed tomography; *aTSA*, anatomic total shoulder arthroplasty; *rTSA*, reverse total shoulder arthroplasty; *NAV*, navigated planned arthroplasties; *PLA*, planned arthroplasties.

In total, we collected data from 197 patients, covering 205 shoulder arthroplasties (Fig. 1). This population was separated into 2 groups according to the use of intraoperative navigation during surgery.

The study was approved by the institutional review board (IRB-MTP_2023_01_202201259), and written patient consent was systematically obtained. The authors followed the Strengthening the Reporting of Observational Studies in Epidemiology guidelines in writing the study.

### Demographics

The following demographic data were collected from patient records: date of birth, age at surgery, history related to the operated shoulder, etiology, operated side, and dominant side. The populations of planned and navigated arthroplasties were comparable at inclusion.

### Planning procedure

Preoperative planning was carried out using the Equinoxe Planning App software (Exactech, Gainesville, FL, USA). Prior to surgery, the patient was asked to undergo a preoperative morphological assessment using a CT scan of the shoulder, including the entire scapula.

The images obtained were extracted and sent to the laboratory via the software. Using the software, a technician generated a 3D model of the scapula, determined the Friedmann line,^4^ and the 3D orientation of the native glenoid plane.

Planning of the glenoid implant was then carried out by the surgeon directly on the software, enabling the choice of implant adapted to the indication (anatomical or inverted total arthroplasty, standard or augmented implant), and its planned positioning in space.

The planning process had several objectives. In the case of aTSA, the aim was to restore anatomy. In this way, a minor deformity could be tolerated but not fully corrected at the planning stage. For the rTSA, the aim was to approach a “neutral” positioning of the glenoid implant, at 0° of version and inclination.

The morphology of the glenohumeral joint (according to Walch's classification^21^), the native and planned version and inclination values, the planned implant type and size, and the expected contact surface between implant and bone were collected.

### Surgical technique

The procedure was performed via a deltopectoral approach, allowing access to the coracoid. Additional navigation steps beyond standard instrumentation included fixing a tracker to the coracoid with two small threaded pins, recording system orientation by probing preselected points on the glenoid and scapula, and recording the final position of the implanted glenoid component. In the event of failure of the navigation procedure (unavailable equipment, failed coupling with the station, sensor migration, aberrant intraoperative data, etc.), the patient was integrated into the planned group.

### Postoperative computed tomography evaluation

A postoperative CT scan was systematically performed. Acquisition included the entire scapula and was performed according to a metal artifact–attenuation protocol. The images were integrated into the software to produce a 3D model in the same way as the preoperative. Based on this, we collected the glenoid implant positioning parameters (version, inclination, measured contact surface) after surgery.

### Outcome criteria

The primary endpoint was a significant difference in glenoid implant positioning postoperatively, compared with preoperative planning, in the navigated and planned arthroplasty groups.

For the secondary endpoints, each study included a subgroup analysis based on arthroplasty type to look for differences in implant positioning, as well as an analysis focusing specifically on significant glenoid deformities.

### Statistical evaluation

Statistical analyses were performed using EasyMedStat statistical analysis software (version 3.27; EasyMedStat, Levallois-Perret, France). Data normality was assessed using the Shapiro test for quantitative variables. Categorical variables (expressed as numbers and percentages) were compared using the chi-square test or Fisher's exact test, depending on whether the conditions for each test were met by the data. Continuous data with a normal distribution were presented as means and standard deviations, or as median and ranges otherwise. Pre- and postoperative comparisons for paired samples were performed using the paired Student's t-test for normally distributed data, and the Mann–Whitney test otherwise. The significance level was set at *P* < .05.

## Results

### Initial characteristics

After the exclusion of patients with missing data, 153 TSA were included and 101 (67%) benefited from intraoperative navigation. The study population and initial characteristics are summarized in Table I. There were no differences at inclusion between each group, based on demographic, type of implants, or glenoid deformity.

**Table I:** Initial characteristics study of glenoid implant positioning.

Initial characteristics	Total population	NAV group	PLA group
Nb. of cases	153	101 (66%)	52 (34%)
Sex ratio (M/F)	49/104 (32%/68%)	29/72 (28%/72%)	20/32 (39%/64%)
Age	72.5 (±8.2)	72.0 (±7.8)	73.5 (±8.8)
Operated side (right)	94 (62%)	65 (65%)	29 (57%)
Type of arthroplasty			
aTSA	50 (33%)	38 (38%)	12 (23%)
rTSA	103 (67%)	63 (62%)	40 (77%)
Walch proportion (%)		A1 :43 (42%)	A1 :22 (42%)
		A2: 26 (26%)	A2: 13 (26%)
		B1 :16 (16%)	B1: 8 (16%)
		B2: 13 (13%)	B2: 7 (13%)
		B3 :3 (3%)	B3: 1 (1%)
		C : 0%	C : 1 (1%)

*NAV*, navigated planned arthroplasties; *PLA*, planned arthroplasties; *rTSA*, reverse total shoulder arthroplasty; *aTSA*, anatomic total shoulder arthroplasty.

No significant differences at inclusion were observed in the 2 groups. Reasons for exclusion included:

- Absence of data from preoperative planning software (11 arthroplasties).
- Failure to perform a postoperative CT scan (34 arthroplasties).
- Absence of pre- and postoperative data (7 arthroplasties).

Regarding native and planned glenoid characteristics (Table II), the mean native glenoid version was higher in the navigated arthroplasty group compared with the planned group (−9.2° (±7.9) vs. −6.5° (±10.7), *P* = .072). Mean glenoid inclination was similar in both groups (2.3° (±6.3) vs. 3.0° (±6.5), *P* = .54). The two groups were also comparable at inclusion according to Walch classifications. Planification assessed in each group was comparable.

**Table II:** Comparison of preoperative and planned glenoid implant positioning.

Initial characteristics	NAV group	PLA group	*P* value
Native version (°)	−9.2 (±7.9)	−6.5 (±10.7)	.072
Native inclination (°)	2.3 (±6.3)	3.0 (±6.5)	.54
Planned version (°)	−1.9 (±3.2)	−1.7 (±4.3)	.916
Planned inclination (°)	0.5 (±2.2)	0.5 (±2.1)	.665

*NAV*, navigated planned arthroplasties; *PLA*, planned arthroplasties.

### Can intraoperative navigation improve glenoid implant positioning?

In our population, we found no significant difference in planned implant position (Table III). The postoperative version measured was −2.5° (±4.7°) in the NAV group and −1.6° (±6.2°) in the PLA group (*P* = .259). The measured postoperative inclination was 3.8° (±4.9°) in the NAV vs. 2.0° (±8.1°) in the PLA group (*P* = .312). No significant differences were found.

**Table III:** Comparison of postoperative glenoid implant positioning and difference in planning and postoperative positioning between the groups.

Postoperative positioning	NAV group	PLA group	*P* value
Postoperative version (°)	−2.5 (±4.7) CV = 188%	−1.6 (±6.2) CV = 388%	.259
Postoperative inclination (°)	3.8 (±4.9) CV = 129%	2.0 (±8.1) CV = 405%	.312
Planification-postoperative deviation (version) (°)	3.0° (±2.9) CV = 97%	3.7° (±3.0) CV = 81%	.157
Planification-postoperative deviation (inclination) (°)	4.2° (±3.5) CV = 83%	6.5° (±5.1) CV = 78%	.004∗

*NAV*, navigated planned arthroplasties; *PLA*, planned arthroplasties; *CV*, coefficient of variation.

∗ *P*-value lower than .05.

In the NAV group, we found a significant decrease in the postoperative deviation from the inclination planning compared to the PLA group (4.2° (±3.5) vs. 6.5° (±5.1), *P* = .004), without, however, finding differences in the version deviation.

**Figure 2:**
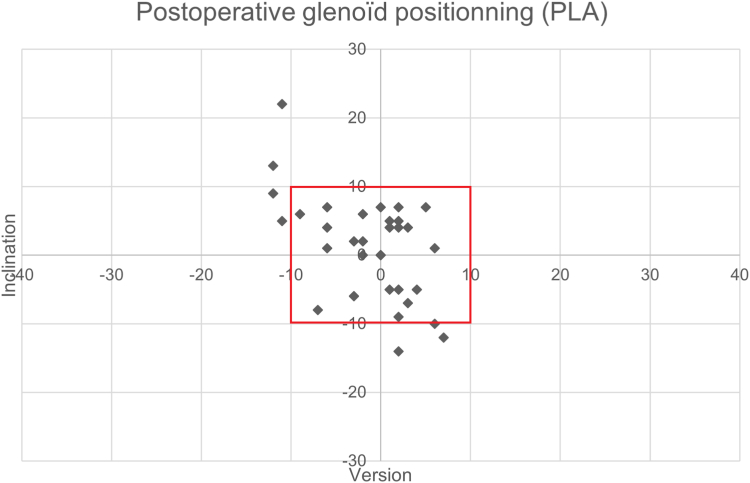
Postoperative positioning in version and inclination of TSA in the NAV and PLA groups. *TSA*, total shoulder arthroplasty; *NAV*, navigated planned arthroplasties; *PLA*, planned arthroplasties.

**Figure 3:**
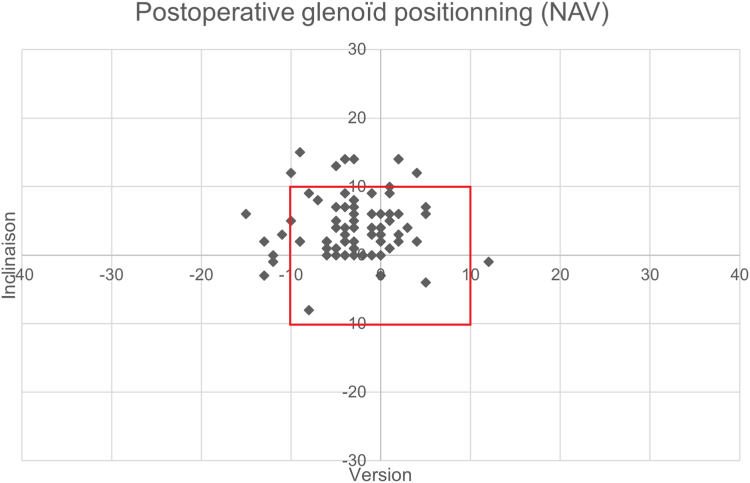
Postoperative positioning in version and inclination of TSA in the NAV and PLA groups. *TSA*, total shoulder arthroplasty; *NAV*, navigated planned arthroplasties; *PLA*, planned arthroplasties.

We measured the coefficient of variation for each of these parameters, to measure the dispersion of the mean positioning of the glenoid implant in each of the groups. We found, in the NAV group, a decrease in the coefficient of variation, suggesting a lower dispersion of the mean positioning of the implant when using navigation. Figures 2 and 3 illustrate the postoperative positioning of each prosthesis in each of the groups.

### Does navigation allow better positioning in cases of significant glenoid deformation?

In this subgroup study, we included all TSA with a native glenoid deformity of more than 10°, according to version and inclination. Seventy-one TSA had a native glenoid version of more than 10°, and 29 a native inclination of more than 10°. In these subgroups, we looked for an increase in the difference between planned and postoperative positioning of the glenoid implant, depending on navigation and initial deformity. The data are summarized in Table IV.

**Table IV:** Difference in glenoid implant positioning compared with preoperative planning in significant version and tilt deformities.

	NAV group	PLA group	*P* value
Native version >|10°|			
Number of cases	48	23	
Postoperative version (°)	−4.1 (±4.9)	−4.4 (±6.8)	.522
Postoperative inclination (°)	3.6 (±5.1)	5.8 (±8.2)	.175
Planification postoperative deviation (version) (°)	3.0 (±3.0)	3.8 (±2.9)	.122
Planification postoperative deviation (inclination) (°)	4.3 (±3.7)	8.0 (±6.3)	.014∗
Native inclination >|10°|			
Number of cases	19	10	
Postoperative version (°)	−2.5 (±4.3)	−0.5 (±8.6)	.222
Postoperative inclination (°)	5.0 (±4.4)	2.2 (±8.3)	.871
Planification postoperative deviation (version) (°)	3.1 (±2.8)	3.2 (±1.7)	>.442
Planification postoperative deviation (inclination) (°)	4.3 (±3.3)	6.7 (±6.3)	.354

*NAV*, navigated planned arthroplasties; *PLA*, planned arthroplasties.

∗ *P*-value lower than .05.

When analyzing significant native deformations in version, we found a significant decrease in the postoperative deviation from the planning in inclination, when using intraoperative navigation (4.3° vs. 8.0°, *P* = .014). However, we did not find any difference regarding the deviation from the planning in version, as well as on the average postoperative positioning of the implants.

Regarding significant native deformations in inclinations, we found no significant difference in the mean positioning of the glenoid implant. We also found no difference regarding the deviation from planning.

### Is there a difference in glenoid implant positioning according to the type of arthroplasty?

A total of 103 rTSA and 50 aTSA were analyzed. We included in the NAV group 63 rTSA and 38 aTSA, and in the PLA group 40 rTSA and 12 aTSA.

Concerning rTSA (Table V), the two groups were comparable at inclusion, except for the native version (respectively −8.3° in the NAV group vs. −5.3°, *P* = .05). However, there was a difference in implant placement, with augmented implants used in 50.8% of cases in the NAV group vs. 10.0% in the PLA group (*P* < .001).

**Table V:** Comparative characteristics of navigated and planned rTSA.

rTSA	NAV group	PLA group	*P* value
Native version (°)	−8.4 (±8.0)	−5.4 (±10.7)	.035∗
Native inclination (°)	2.9 (±6.2)	3.2 (±6.3)	.811
Planned version (°)	−1.2 (±2.6)	−1.3 (±4.6)	.607
Planned inclination (°)	0.1 (±1.7)	0.1 (±1.6)	.965
Postoperative version (°)	−1.7 (±4.2)	−0.9 (±6.3)	.268
Postoperative inclination (°)	3.4 (±4.0)	−1.1 (±8.7)	.121
Planification postoperative deviation (version) (°)	2.7 (±2.5)	3.9 (±3.1)	.047∗
Planification postoperative deviation (inclination) (°)	3.9 (±3.4)	7.1 (±5.1)	<.001∗
Augmented implant (%)	32 (50.8%)	4 (10.0%)	<.001∗

*rTSA*, reverse total shoulder arthroplasty; *NAV*, navigated planned arthroplasties; *PLA*, planned arthroplasties.

∗ *P*-values lower than .05.

No significant difference was found postoperatively regarding the average postoperative positioning of the glenoid implant between the two groups. However, we found a significant reduction in the postoperative deviation from planning in the NAV group, in version (2.7° (±2.5) vs. 3.9° (±3.1), *P* = .047) and in inclination (3.9° (±3.4) vs. 7.1° (±5.1), *P* < .001).

In the aTSA group, no difference was found in the NAV group compared with the PLA group. No significant differences in positioning or implant were found, either during planning or postoperatively. Characteristics are summarized in Table VI.

**Table VI:** Comparative characteristics of navigated and planned aTSA.

aTSA	NAV group	PLA group	*P* value
Native version (°)	−10.4 (±7.8)	−10.3 (±10.6)	.99
Native inclination (°)	1.3 (±6.3)	2.3 (±7.5)	.624
Planned version (°)	−3.2 (±3.7)	−3.3 (±3.0)	.872
Planned inclination (°)	1.3 (±2.6)	1.9 (±3.0)	.692
Postoperative version (°)	−3.9 (±5.1)	−4.1 (±5.5)	.902
Postoperative inclination (°)	4.3 (±6.1)	4.9 (±5.0)	.811
Planification postoperative deviation (version) (°)	3.5 (±3.5)	3.0 (±2.8)	.662
Planification postoperative deviation (inclination) (°)	4.7 (±3.7)	4.7 (±4.6)	.673
Augmented implant (%)	8 (21.1%)	4 (33.3%)	.448

*aTSA*, anatomic total shoulder arthroplasty; *NAV*, navigated planned arthroplasties; *PLA*, planned arthroplasties.

### Influence of navigation on glenoid implant malposition

We conducted a study to investigate whether navigation could reduce the proportion of implant malposition. This criterion was defined by a positioning version or inclination of more than 10° from the “neutral” glenoid plane or by a postoperative difference of more than 10° in version or tilt compared with planning.

The data are summarized in Table VII. In the NAV group, we found a nonsignificant reduction in the proportion of implants positioned more than 10° from neutral compared with the PLA group.

**Table VII:** Comparison of glenoid implant positioning errors during navigated and PLA.

Implant misplacement	Nb. of cases (% of total)	NAV group	PLA group	*P* value
Version >|10°|	14 (9.2%)	7 (6.9%)	7 (13.5%)	.237
Inclination >|10°|	20 (13.1%)	10 (9.9%)	10 (19.3%)	.13
Planification postoperative deviation (version) >|10°|	5 (3.3%)	3 (3.0%)	2 (3.9%)	.999
Planification postoperative deviation (inclination)>|10°|	13 (8.5%)	5 (5.0%)	9 (17.3%)	.017∗

*NAV*, navigated planned arthroplasties; *PLA*, planned arthroplasties.

∗ *P*-value lower than .05.

We also found a significant reduction in the number of prostheses with a significant planning deviation in inclination (5.0% vs. 17.3%, *P* = .017). However, this reduction was not observed for version deviations.

## Discussion

Our study found no significant differences in mean glenoid implant positioning using intraoperative navigation compared with preoperative planning only. However, a decrease in the proportion of implants mispositioned was observed in the navigated prosthesis group.

Several studies have compared intraoperative navigation with other techniques to assist implant positioning, and most of those reporting significant results of navigation on glenoid implant positioning found a difference in positioning of the order of 5° compared with the planned implant position.

Nevertheless, few studies of navigation use a scanographic measurement of postoperative implant positioning via a systematic postoperative CT scan.

Jones et al^8^ found better postoperative positioning of the glenoid implant when using navigation compared with the freehand technique, with a difference from the planned version of 1.9° and from the planned inclination of 2.4° (compared with a difference of 5.9° and 6.3°, respectively, from planning in the nonnavigated group). This is similar to the results found by Kida et al^9^ in a study of 71 navigated rTSA, who found a mean postoperative version of 0.2° in the navigated group vs. −2.0° in the nonnavigated group and a superior inclination of 0.3° vs. 2.4°, with a significant difference. Nashikkar et al^15^ found similar glenoid implant positioning with and without navigation. However, the use of navigation reduced the variability of glenoid implant positioning between patients, although a positioning error rate of over 10% on implant inclination was found in both groups. Larose et al^11^ found a reduction in the incidence of aberrant positioning of the glenoid implant thanks to the live display of the drilling depth, the orientation of the glenoid base, as well as the optimization of the positioning of the screws.

However, navigation would provide better implant positioning than patient-specific instrumentation or the freehand technique.^23^

Planned glenoid implant positioning plays an important role during navigated surgery, helping the surgeon to reproduce this planning. Parsons et al^16^ studied intra- and interobserver variability in preoperative planning, solely on aTSA. Final planned implant positioning showed a mean version of −1.4° and a mean final inclination of 2.1°. This study showed poor reproducibility in terms of planned version, implant type (augmented or non-augmented), and planned implant size, suggesting that several solutions can be proposed to achieve a similar postoperative reconstruction. However, a retroversion of 10° should not be exceeded.^3^^,^^7^^,^^13^

The use of augmented implants can provide a solution in cases of significant glenoid deformity. In our study, we also used a combination of asymmetrical milling and augmented implants for large deformities, especially for rTSAs.

Thus, peroperative navigation plays an important role in the selection of implants. In our study, the use of navigation was associated with an increase in the placement of augmented implants. This is also found in the literature. In their study involving 51 navigated reverse arthroplasties, Sprowls et al^18^ used an augmented implant in 76.5% of cases, compared to 19.1% in the context of nonnavigated arthroplasties. This result is found in other studies.^14^^,^^17^^,^^22^^,^^23^ One explanation could be that some surgeons, given the difficulties caused by posterior humeral subluxation and the deformation of the native glenoid, tend to preferentially use navigation assistance in more complex cases and to “limit themselves” to preoperative planning alone in simpler cases.

Some authors report a decrease in postoperative complications following its use. Thus, it has been observed^5^^,^^23^ a reduction in hospital stays, a decrease in secondary rotator cuff tears in the context of anatomical arthroplasties, the rate of prosthetic dislocation, scapular notching, and a lower incidence of prosthetic revisions, regardless of the cause. However, a hindrance to the use of navigation remains the failure of the procedure, forcing the surgeon to revert to a conventional technique intraoperatively. Kircher et al^10^ reported a failure rate of the procedure of 37%. This rate, however, seems to vary with the user's experience, with Youderian et al^23^ reporting a success rate of 99.9% in a study involving 17 surgeons accustomed to this procedure, and Larose et al^11^ reporting a rate of 98%. In our study, navigation failures during the procedure were classified as controls, and among the reports, 27 nonnavigated patients were categorized as failures of the procedure during the intervention, resulting in a failure rate of 17.3%.

There are several limitations to our study. The distribution between the navigated and planned TSA groups was not randomized. Nevertheless, the sizes of the two populations remain homogeneous and comparable. Regarding postoperative reconstructions, the presence of metallic or reconstruction artifacts may limit the reconstruction of the Friedman line and the plane of the glenoid.

## Conclusion

Our study found similar results regarding the use of intraoperative navigation associated with preoperative planning, compared to planning alone, on the positioning of the glenoid implant. We highlighted a decrease in the deviation from planning in the context of rTSAs, as well as a trend towards a decrease in aberrant glenoid positioning when using intraoperative navigation.

The use of computer-assisted navigation and surgery will continue to evolve, which may provide additional opportunities for future improvements. However, further studies are needed to determine the long-term radiographic and clinical outcomes associated with intraoperative navigation.

## Disclaimers:

Funding: No funding was disclosed by the authors.

Conflicts of interest: Bertrand Coulet reports a relationship with Exatech France S.A.S. that includes: consulting or advisory. The other authors, their immediate families, and any research foundation with which they are affiliated have not received any financial payments or other benefits from any commercial entity related to the subject of this article.
